# Brain activity and transcriptional profiling in mice under chronic jet lag

**DOI:** 10.1038/s41597-020-00709-6

**Published:** 2020-10-21

**Authors:** Qian Gao, Suliman Khan, Luoying Zhang

**Affiliations:** 1 Key Laboratory of Molecular Biophysics of Ministry of Education, College of Life Science and Technology, Huazhong University of Science and Technology, Wuhan, Hubei 430074 China; 2 Institute of Brain Research, Huazhong University of Science and Technology, Wuhan, Hubei 430074 China; 3 Department of Cerebrovascular Diseases, The Second Affiliated Hospital of Zhengzhou University, Zhengzhou, Henan 450014 China

**Keywords:** Transcriptomics, Circadian rhythms and sleep

## Abstract

Shift work is known to be associated with an increased risk of neurological and psychiatric diseases, but how it contributes to the development of these diseases remains unclear. Chronic jet lag (CJL) induced by shifting light-dark cycles repeatedly is a commonly used protocol to mimic the environmental light/dark changes encountered by shift workers. Here we subjected wildtype mice to CJL and performed positron emission tomography imaging of glucose metabolism to monitor brain activities. We also conducted RNA sequencing using prefrontal cortex and nucleus accumbens tissues from these animals, which are brain regions strongly implicated in the pathology of various neurological and psychiatric conditions. Our results reveal the alterations of brain activities and systematic reprogramming of gene expression in brain tissues under CJL, building hypothesis for how CJL increases the susceptibility to neurological and psychiatric diseases.

## Abstract (summary)

**Table:** 

Measurement(s)	brain measurement • sleep duration • sleep quality • fragments per kilobase of exon per million mapped reads
Technology Type(s)	positron emission tomography • animal activity monitoring system • RNA sequencing
Factor Type(s)	chronic jet lag
Sample Characteristic - Organism	Mus musculus
Sample Characteristic - Environment	laboratory environment

Machine-accessible metadata file describing the reported data: 10.6084/m9.figshare.12924248

## Background & Summary

Approximately 15%–30% of the working population worldwide are engaged in some type of shift work^1^. Based on epidemiological surveys, shift work is associated with numerous adverse health outcomes such as type 2 diabetes, cancer, cardiovascular disorders and immune dysfunction^2,3^. Shift work is also known to increase the risk of neurological diseases. To be specific, shift work may trigger migraine, exacerbate epilepsy and increase dementia incidence^4–6^. In addition, shift work is associated with cognitive deficits^7–9^, sleep disorders^1,10,11^, mood alteration and increases the risk of mental illnesses including depression, anxiety, alcohol abuse, and schizophrenia^12–15^. Individuals undergoing shift work experience alternating light/dark cycles for extended time and suffer from circadian disruptions due to the misalignment between endogenous rhythm and external time. To mimic shift work condition, a number of studies used rodents subjected to chronic jet lag (CJL) by continuously shifting the timing of light-dark cycles and found that CJL promotes tumour progression^16–18^, obesity^19^, addiction^20^, and impairs innate immune responses^21^. Recent work in rodents also demonstrated that CJL leads to phenotypes related to mood disorders, but the mechanism is largely unknown^22,23^.

Glucose turnover rate serves as an indicator of brain activities, while alterations of brain activities are linked to many neurological and psychiatric diseases^24^. Abnormal glucose metabolism in the brain has been reported in dementia, epilepsy, major depressive disorder, and bipolar disorder^25–27^. The prefrontal cortex (PFC) is known to be involved in regulating a number of cognitive and emotional processes^28^. Dysfunction of the PFC has been found in various psychiatric and neurological disorders, including depression, anxiety disorders, addiction, schizophrenia, autism spectrum disorders, Alzheimer’s disease, and Parkinson’s disease^29–32^. Nucleus accumbens (NAc) plays a central role in processing motivation, reward and aversion^33–35^. NAc has been implicated in the pathophysiology and treatment of mental illnesses such as major depressive disorder, addictive disorders, schizophrenia, obsessive-compulsive disorder and anorexia nervosa^36–39^.

**Fig. 1:**
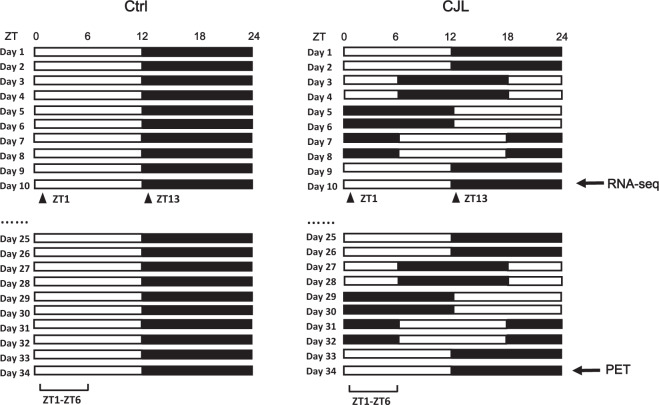
Light regime for chronic jet lag treatment. Male wildtype mice were exposed to stable or shiftwork-like light conditions. Black bars represent dark phase and hollow bars represent light phase. Solid triangles and square brackets indicate sampling time of RNA-seq and time of PET imaging, respectively. Ctrl, control; CJL, chronic jet lag.

**Fig. 2:**
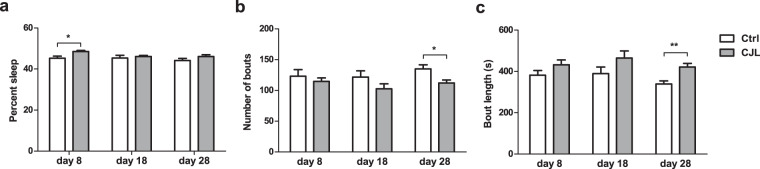
Chronic jet lag does not reduce sleep duration. Average percent sleep (**a**), the number of sleep bout in 24 hours (**b**) and average bout length in seconds (**c**) of male wildtype mice on the 8^th^, 18^th^, and 28^th^ day of CJL (N = 7). Data are presented as mean ± SEM, Mann-Whitney test, *P < 0.05, **P < 0.01. Ctrl, control; CJL, chronic jet lag.

**Fig. 3:**
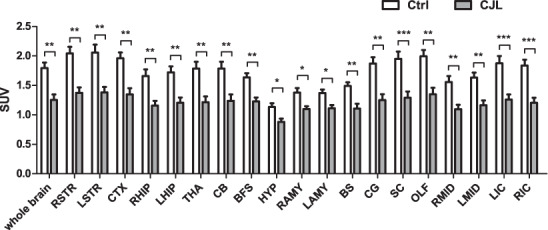
Chronic jet lag reduces brain glucose metabolism. Bars indicate standard uptake value (SUV) of glucose in the entire brain and different brain regions of control mice (N = 6) and mice on the 34^th^ day of CJL treatment (N = 9). Data are presented as mean ± SEM, Mann-Whitney test, *P < 0.05, **P < 0.01, ***P < 0.001. Ctrl, control; CJL, chronic jet lag; RSTR, right striatum; LSTR, left striatum; CTX, cerebral cortex; RHIP, right hippocampus; LHIP, left hippocampus; THA, thalamus; CB, cerebellum; BFS, basal forebrain septum; HYP, hypothalamus; RAMY, right amygdale; LAMY, left amygdale; BS, brain stem; CG, central gray; SC, superior colliculus; OLF, olfactory bulb; RMID, right midbrain; LMID, left midbrain; LIC, left inferior colliculus; RIC, right inferior colliculus.

In this study, we subjected wildtype mice to CJL following a previously reported protocol^40^ (Fig. 1). Total sleep duration is not reduced by CJL, indicating that the physiological and molecular changes we report here are not due to deficient sleep caused by CJL (Fig. 2). We employed positron emission tomography (PET) to monitor glucose uptake in the brain of mice maintained under baseline condition or CJL for slightly over a month, as it has previously been shown that CJL treatment for about a month can elicit prominent changes in metabolic indices and lung mechanics^40,41^. We present raw PET images as well as quantified data collected on Day 34 of CJL treatment, which demonstrate that CJL significantly reduces activities throughout the brain (Fig. 3). Given the prominent and pervasive alterations in brain activity observed by Day 34 of CJL, we reasoned that molecular changes contributing to this should start to occur at an earlier stage of CJL treatment. It has been reported that metabolic phenotypes start to become significant by Day 10 of CJL, and ten days of CJL is sufficient to induce substantial effects on the expression of a number of circadian genes^40,42^. Therefore, we conducted RNA sequencing (RNA-seq) on PFC and NAc harvested from animals during the light and dark phase under baseline condition and on Day 10 of CJL treatment, respectively. These data will provide valuable information for studies that investigate how shift work or circadian disruptions increase the risk of disorders associated with the brain.

## Methods

### Animals and chronic jet lag treatment

8-week-old male C57BL/6 J mice purchased from Model Animal Research Center of Nanjing University were assigned to different cages randomly with approximately 5 animals per cage. Animals were housed in light-tight cabinets with time-controlled illumination. Food and sterilized water were accessible *ad libitum*. The mice were housed under 12 hour light: 12 hour dark (12L12D) for 2 weeks for entrainment prior to CJL treatment. The experimental design is displayed in Fig. 1. For CJL group, lights on and off times were advanced by 6 hours every 2 days following a previously published protocol, whereas for control group they remain unchanged^40,43^. Nine mice were subjected to PET imaging on Day 34 of CJL (the last jetlag event before PET imaging occurred on Day 32) along with six controls. The brain tissues of six mice (three per time point) were harvested for RNA-seq on Day 10 of CJL (the last jetlag event before tissue collection occurred on Day 8) along with equal number of controls. To assess sleep, seven mice were individually housed and sleep monitoring was conducted on Day 8, 18, and 28 of CJL. All mouse work was performed in accordance with the guidelines of Institutional Animal Care and Use Committee at Huazhong University of Science and Technology.

### Sleep monitoring

Sleep was monitored by PiezoSleep Mouse Behaviour Tracking System as described earlier^44^. Briefly, seven animals were individually housed in a cage with piezoelectric film sensor underneath that transmitted activity signals (motions and breathing movements) to monitoring software (PiezoSleep 2.18, Signal Solutions). Sleep can be distinguished from wakefulness by rhythmic signals (approximately 3 Hz) generated by typical respiration patterns during sleep^44^. The baseline pressure signal from the piezoelectric sensors was sampled at 120 Hz. Features associated with sleep and wake behaviors were extracted from the pressure signals and saved every 2 seconds. Sleep-wake classification was performed automatically based on decision statistics^45^. Data collected were binned over 1 hour using the average of percent sleep (average percent of time in sleep state). Data were analyzed with SleepStats and exported as hourly percent sleep, hourly sleep bout number and hourly average sleep bout length of the specified day. Sleep bout was computed as the duration of an interval of continuous sleep. The minimum sleep bout length parameter was set to 30 s by default, which means a bout length count was initiated only when a 30 s-interval contained greater than 50% sleep^46,47^. Setting the minimum bout length as 30 s can eliminate the impact of short and ambiguous arousals on computing bout length and reduce the probability of error^46^. Based on the exported data, average percent sleep, sleep bout number, and average length of sleep bout of a specified day were calculated.

### PET

^18^F-fluorodeoxyglucose (^18^F-FDG) is the most commonly used marker for glucose metabolism in PET imaging^48^. Nine male C57BL6/J mice subjected to CJL and six control mice were used. Mice were fasted during the dark phase for 12 h before the test. Prior to PET imaging, animals were injected with (250 ± 10 μCi) ^18^F-FDG intraperitoneally. After one hour, they were anesthetized with 2% isoflurane and imaging was conducted from Zeitgeber Time 1 (ZT1, 1 hour after lights on; ZT0 is defined as the time of lights on) to ZT6. Images of mouse brains were obtained with the static scanning pattern (10 min) by Trans-PET®BioCaliburn® LH (Raycan Technology, China), a PET system for imaging small animals^49^. The PET images were reconstructed using three-dimensional OSEM method with a voxel size of 0.5 × 0.5 × 0.5 mm^3^. Glucose uptake was measured as the mean standardized uptake value (SUV).

### Brain dissection and RNA extraction

Brain tissues of three CJL treated animals and three control animals fed *ad libitum* were harvested at ZT1 and ZT13, respectively. After cervical dislocation, the skin was flipped over the eyes to free the skull. Then the skull was broken and brain was removed gently out of the skull. The brain was immediately transferred to a Petri dish chilled on ice and placed with ventral surface facing up. The brains of mice were dissected according to previously published protocols^50–53^. Coronal sections were made from the rostral end of the brain using a sharp and chilled razor blade. PFC, the anterior part of the brain just behind the olfactory bulb, was removed from the first ~1.5 mm-thick coronal section^50,51^. NAc was located in the subsequent section (~1.0 mm-thick) and was identified based on the location of anterior commissure with the approximate anterior-posterior coordinate of +1.8 mm~0.6 mm from Bregma^52,53^. The dissected PFC and NAc tissues were immediately transferred to liquid nitrogen and stored at −80 °C for further processing.

Total RNA was extracted using RNA isolater (Vazyme, China). RNA integrity was assessed with RNA Nano 6000 Assay Kit of the Bioanalyzer 2100 system (Alilent Technologies, USA) before the samples were sent for RNA-seq.

### Library preparation, RNA sequencing and data processing

The sequencing libraries were generated using VAHTS mRNA-seq v2 Library Prep Kit for Illumina® (Vazyme, NR601) following manufacturer’s recommendations. Briefly, mRNA was purified and fragmented and cDNA was synthesized with the mRNA fragments as templates. Then the cDNAs were ligated with special sequencing adaptor and the products with appropriate size were selected for PCR. After passing quality examination, the generated libraries were sequenced on Illumina Hiseq X Ten platform with 150 bp paired-end module.

The raw reads were filtered to produce clean data by excluding sequencing adaptors and low quality reads, including reads containing over 5% “N” and those containing >50% bases with quality value less than 10. The quality assessment of clean data was performed using FastQC v0.11.9.

Sequencing reads were then aligned to the mouse mm10 reference genome using HISAT2. The differential expression analysis was performed by DESeq. 2 with default parameters. The significance threshold of *P* < 0.05 was applied. The number of differentially expressed genes (DEGs) and the number of genes that exhibited a fold-change >2 in each brain tissue at each time point were displayed in Table 1.

**Table 1:** Number of DEGs (*P* < 0.05) and genes that exhibit more than two-fold change (FC > 2 or <0.5).

		No. of DEGs	No. of up-regulated genes	No. of down-regulated genes	No. of genes with *P* < 0.05 & FC > 2	No. of genes with *P* < 0.05 & FC < 0.5
**PFC**						
ZT1	CJL vs Ctrl	3088	1535	1553	194	240
ZT13	CJL vs Ctrl	2020	1189	831	139	204
Ctrl	ZT13 vs ZT1	4699	2211	2488	278	304
CJL	ZT13 vs ZT1	1221	573	648	162	253
**NAc**						
ZT1	CJL vs Ctrl	2569	1470	1099	507	212
ZT13	CJL vs Ctrl	2590	850	1740	207	358
Ctrl	ZT13 vs ZT1	3169	2062	1107	528	225
CJL	ZT13 vs ZT1	1832	886	946	202	267

Ctrl, control; CJL, chronic jet lag.

## Data Records

Sequencing data and the list of differentially expressed genes can be accessed at NCBI Gene Expression Omnibus (GEO) with the accession number GSE153540^54^. The PET data and images have been deposited in Figshare^55^.

## Technical Validation

**Fig. 4:**
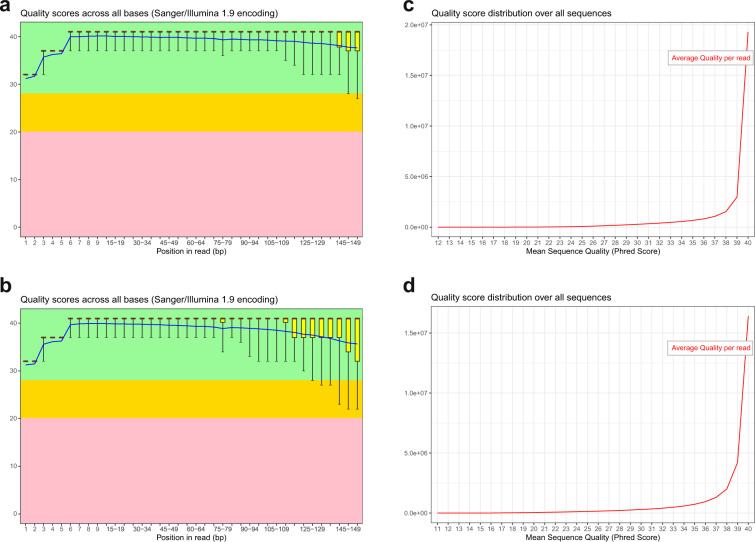
FastQC report for the assessment of RNA sequencing quality. (**a**,**b**) The quality scores per base along the reads from one sample. The yellow box represents the interquartile range (25–75%). The upper and lower whiskers represent the 90% and 10% points, respectively. The blue line indicates mean value. (**c**,**d**) The distribution of mean sequence quality scores for all reads from one sample.

A high percentage of clean reads from each sample was acquired after filtering the raw reads. The statistics summary of clean reads is presented in Table 2. All samples produced more than 98.5% clean reads and >93.60% of the clean data were mapped to the reference genome. In addition, GC content, Q20 and Q30 were also calculated. For all samples, the GC content was stable with distributed range from 48.36% to 49.18%. The read base quality was assessed by FastQC and displayed in Fig. 4. Most of the base quality scores were above 30, suggesting high-quality RNA-seq data.

**Table 2:** Statistics of sequencing data.

Samples	Raw reads	Clean reads	Clean read rate (%)	GC (%)	mapping rate (%)	Q20 (%)	Q30 (%)	Accession
PFC_ZT1_Ctrl_1	59674380	59285246	99.35	48.75	96.35	97.28	93.32	GSM4646833
PFC_ZT1_Ctrl_2	59262454	58394418	98.54	48.36	96.91	97.63	93.98	GSM4646834
PFC_ZT1_Ctrl_3	53657490	53181490	99.11	48.65	96.54	97.33	93.41	GSM4646835
PFC_ZT13_CJL_1	52488284	52184492	99.42	49.18	96.32	97.12	92.99	GSM4646836
PFC_ZT13_CJL_2	54765670	54529024	99.57	48.81	96.74	97.06	92.89	GSM4646837
PFC_ZT13_CJL_3	51643852	51358972	99.45	48.89	96.50	96.97	92.72	GSM4646838
PFC_ZT1_CJL_1	55064574	54814420	99.55	49.06	96.65	97.09	92.94	GSM4646839
PFC_ZT1_CJL_2	56191198	55940284	99.55	49.09	96.77	97.00	92.77	GSM4646840
PFC_ZT1_CJL_3	55392450	55117524	99.50	49.34	96.70	97.18	93.09	GSM4646841
PFC_ZT13_Ctrl_1	55479172	55225860	99.54	49.06	96.80	97.19	93.14	GSM4646842
PFC_ZT13_Ctrl_2	51733648	51449378	99.45	48.84	96.78	97.17	93.1	GSM4646843
PFC_ZT13_Ctrl_3	54609704	54328066	99.48	49.08	96.63	97.25	93.27	GSM4646844
NAc_ZT1_Ctrl_1	52250318	52046014	99.61	48.57	93.60	94.72	88.17	GSM4646845
NAc_ZT1_Ctrl_2	46872158	46551084	99.32	48.18	94.76	95.58	89.94	GSM4646846
NAc_ZT1_Ctrl_3	47492160	47295374	99.59	48.94	94.27	95.32	89.42	GSM4646847
NAc_ZT13_CJL_1	49204480	49004402	99.59	49.27	94.22	95.30	89.34	GSM4646848
NAc_ZT13_CJL_2	49269340	49052430	99.56	48.99	93.78	95.03	88.87	GSM4646849
NAc_ZT13_CJL_3	48143562	47907922	99.51	48.92	94.20	95.42	89.62	GSM4646850
NAc_ZT1_CJL_1	54553374	54269114	99.48	48.73	94.28	95.53	89.84	GSM4646851
NAc_ZT1_CJL_2	49040458	48755274	99.42	49.21	94.08	95.36	89.5	GSM4646852
NAc_ZT1_CJL_3	48019432	47811708	99.57	49.05	94.00	95.04	88.87	GSM4646853
NAc_ZT13_Ctrl_1	45860414	45545344	99.31	47.90	95.02	95.72	90.16	GSM4646854
NAc_ZT13_Ctrl_2	52558094	52350316	99.60	49.39	94.42	95.40	89.55	GSM4646855
NAc_ZT13_Ctrl_3	51288372	51117430	99.67	49.50	94.33	95.30	89.38	GSM4646856

Clean data rate = 100% × Clean reads/Raw reads. Ctrl, control; CJL, chronic jet lag.
